# Exploring the Role of Relish on Antimicrobial Peptide Expressions (AMPs) Upon Nematode-Bacteria Complex Challenge in the Nipa Palm Hispid Beetle, *Octodonta nipae* Maulik (Coleoptera: Chrysomelidae)

**DOI:** 10.3389/fmicb.2019.02466

**Published:** 2019-10-31

**Authors:** Nafiu Bala Sanda, Bofeng Hou, Abrar Muhammad, Habib Ali, Youming Hou

**Affiliations:** ^1^State Key Laboratory of Ecological Pest Control for Fujian and Taiwan Crops, Department of Plant Protection, Fujian Agriculture and Forestry University, Fuzhou, China; ^2^Department of Crop Protection, Faculty of Agriculture, Bayero University Kano, Kano, Nigeria; ^3^Department of Entomology, College of Plant Protection, Nanjing Agricultural University, Nanjing, China; ^4^Department of Entomology, University of Agriculture Faisalabad, Okara, Pakistan

**Keywords:** insect immunity, Relish, antimicrobial peptide, *Steinernema carpocapsae*, *Heterorhabditis bacteriophora*, symbiotic bacteria

## Abstract

The humoral immune responses of the nipa palm hispid beetle *Octodonta nipae* involves the inducible expression of the genes coding for antimicrobial peptides (AMPs) which are mediated by immune deficiency signaling pathways. In insects, the nuclear factor-κB (NF−κB) transcription factor, Relish, has been shown to regulate AMP gene expressions upon microbial infections. Here, we dissect the expression patterns of some AMPs in *O. nipae* during infections by entomopathogenic nematodes (EPNs) and their symbionts, before and after Relish knock down. Our results indicate that, prior to gene silencing, the AMPs attacin C1, attacin C2, and defensin 2B were especially expressed to great extents in the insects challenged with the nematodes *Steinernema carpocapsae* and *Heterorhabditis bacteriophora* as well as with their respective symbionts *Xenorhabdus nematophila* and *Photorhabdus luminescens*. The study also established the partial sequence of OnRelish/NF-κB p110 subunit in *O. nipae*, with an open reading frame coding for a protein with 102 amino acid residues. A typical Death domain-containing protein was detected (as seen in *Drosophila*) at the C-terminus of the protein. Phylogenetic analysis revealed that in *O. nipae*, Relish is clustered with registered Relish/NF-κB p110 proteins from other species of insect especially *Leptinotarsa decemlineata* from the same order Coleoptera. Injection of OnRelish dsRNA remarkably brought down the expression of OnRelish and also reduced the magnitude of transcription of attacin C1 and defensin 2B upon *S. carpocapsae* and *H. bacteriophora* and their symbionts infections. Altogether, our data unveil the expression pattern of OnRelish as well as that of some AMP genes it influences during immune responses of *O. nipae* against EPNs and their symbionts.

## Introduction

The alien invasive palm pest *Octodonta nipae* Maulik (Coleoptera: Chrysomelidae) is a hispid beetle of palm plantations which is believed to have originated from Malaysia. It is now the most destructive palm pest in the southern part of China (Hou et al., 2011; Xi et al., 2013; Tang and Hou, 2017; Chen et al., 2018). This pest was spotted for the first time in Hainan Island of Hainan Province as far back as 2001 (Sun et al., 2003; Hou and Weng, 2010; Feng and Hou, 2015). The pest spread to Fujian Province in 2007, where it damages more than 10 palm species belonging to the family Palmae, including *Areca catechu* Linn., *Nypa fruticans* Wurmb, *Washingtonia filifera* (Lindl.) H. Wendl., *Metroxylon sagu* Rottb., *Phoenix canariensis* Chabaud, and so on Li et al. (2014, 2016) and Meng et al. (2016). The pest was reported to mainly attack young leaves of different palm trees, which resulted in significant lost to palm plants industry, city landscaping, and ecological safety (Peng et al., 2018a, b). The immature forms of this pest are gregarious. Adults and larvae heavily attack the middle and unopened epidermal parenchyma which cause the leaflets of the furled fronds to subsequently appear gray-brown and withered, possessing rolled edges, resulting in stunted, and impaired growth that leads to death of the tree (Hua et al., 2014; Zhang et al., 2015, 2017; Ali et al., 2018a, b, 2019). However, this damage restricts the use of insecticides during chemical control of the pest. This warrants the application of biological control agents. The pupae parasitoid *Tetrastichus brontispae* Ferrière (Tang et al., 2014a, b, 2019) was proved to be effective for the control of this pest. Another pathogenic agent is *Metarhizium anisopliae* var. anisopliae (Xu et al., 2011). Additional non-chemical control methods are urgently needed to address the menace of this pest using ecologically friendly alternative methods.

The entomopathogenic nematodes (EPNs) belonging to the families Heterorhabditidae and Steinernematidae are widely used for effective biological control of insect pests. *Heterorhabditis bacteriophora* and *Steinernema carpocapsae* dwell in the soil where they associate with the Gram-negative bacteria *Photorhabdus luminescens* and *Xenorhabdus nematophila* in an obligate and mutualistic relationship, respectively (Hussain et al., 2016). The only stage of the nematodes that survive outside the hosts is the infective juvenile (IJ), which enters the insects by piercing the body wall or via natural openings (Sanda et al., 2018). The IJs release the bacteria into the hemocoel in which they reproduce exponentially, producing different toxins and virulence factors, leading to the death of the insect. These toxins elicit different immune responses. They possess immune-suppressive properties which protect both the nematodes and the bacteria from the counter immune responses of the infected hosts (Jang et al., 2012). However, previous studies also demonstrate that the nematodes also contribute significantly to the pathogenicity of the nematobacterial complex (Han and Ehlers, 2000; Lu et al., 2017; Chang et al., 2019).

In insects, the first step of defense against pathogens like nematodes and their symbiotic bacteria is the recognition of the pathogens, which relies solely on host pattern recognition peptides (Bettencourt et al., 2004). This is triggered by the interaction of hemocyte receptors or plasma proteins with pathogen-associated molecular patterns (PAMPs) like peptidoglycan, lipopolysaccharide and β-1,3-glucan (Buchon et al., 2014). The binding of non-self’s PAMPs on PRPs induces the synthesis of antimicrobial proteins, activates cellular immune responses as well as phenoloxidase cascade reactions, leading to nodule formation, encapsulation, and phagocytosis of the pathogens (Marmaras and Lampropoulou, 2009). However, it has been established recently that *Drosophila* uses a PAMP-independent pathway to recognize some metalloproteases of bacterial origin (Issa et al., 2018). Of the insect immune responses to pathogen invasion, antimicrobial peptides (AMPs) formation is the last line of defense. Their actions take several hours to a few days and are highly specific in their effects. They are synthesized by the fat body and released by hemocytes, gut, salivary glands, ovaries, and midgut (Rosales, 2017). The AMPs are cationic molecules with 8–60 amino acid residues. They are mainly synthesized following immune deficiency (IMD) pathway-dependent signals. AMPs bind to anionic bacterial membranes leading to disruption and cell death (Yi et al., 2014).

Upon pathogen infection, the peptidoglycan recognition protein (PGRP) from the insect host elicits the activation of the IMD pathway through binding to meso-diaminopimelic acid (DAP) -type peptidoglycan. The DAP-type peptidoglycan starts a cascade of activation of Fadd, Dredd, Tak1/Tab2 complex and leads to the activation, translocation and of the nuclear factor-κB (NF−κB) transcription factor, Relish (Erturk-Hasdemir et al., 2009). Relish binds to the promoters of the respective genes to induce AMP gene expressions (Erturk-Hasdemir et al., 2009). This IMD pathway was first discovered in *Drosophila* (Georgel et al., 2001). Relish belongs to the Rel/NF-κB family, which is essential for humoral immune responses in *Drosophila* (Dushay et al., 1996). When Relish mutant was infected, the expression levels of the AMPs was highly reduced compared to wild type, and the animal was highly susceptible to bacterial and fungal infections (Delaney et al., 2006). The symbiotic nematode *H. bacteriophora* was reported to induce the expression of the immune genes encoding AMPs (Brivio et al., 2004; Vega and Kaya, 2012). In another study, *S. carpocapsae* has been shown to cause up-regulation of attacin-A, attacin-B, attacin-C, and drosomycin at 6 and 24 h post infections (Alvandi et al., 2017). Contrarily, Hwang et al. (2013) reported that the expression of AMP gene in insects is inhibited by *X. nematophila* through interfering with eicosanoid signaling. Similarly, in *Spodoptera exigua*, the expression of an AMP gene encoding *Cecropin* was inhibited by *X. nematophila* infection (Ji and Kim, 2004; Duvic et al., 2012). The expression levels of AMPs, however, vary between the nematodes and their symbiotic bacteria. As reported by Darsouei et al. (2017), the mRNA expression levels of attacin, cecropin, and spodoptericin were higher in *S. exigua* larvae treated with *S. carpocapsae* than *H. bacteriophora*. This was supported by Castillo et al. (2015). *Photorhabdus* produces and releases a number of toxins and virulent factors that suppress the immune system through inactivating phenoloxidase system, destroying immune cells, and degrading AMPs. Similar data were also reported by Rahatkhah et al. (2015) where attacin expression level decreased significantly as early as 2 h post *H. bacteriophora* injection. It was shown that *X. nematophila* generally suppresses the immune systems of lepidopterans (*Manduca sexta* and *S. exigua*) (Ji and Kim, 2004; Park et al., 2007) but not *Drosophila* (a dipteran) (Aymeric et al., 2010). This hypothesized that the immune inhibition stage is present in lepidopterans but absent in the dipteran fly, *Drosophila*. Similarly, nematodes and their symbiotic bacterial infections resulted in parallel and fluctuated pattern of AMPs gene expressions in *S. exigua* larvae. However, the expression pattern of AMPs in *O. nipae* larvae upon infection with nematodes in complex with their symbiotic bacteria is not known.

Recently, Meng et al. (2018) exploited the roles of AMP gene expressions on parasitism of *O. nipae*. The study demonstrated that all the AMPs were up-regulated at all time-points, except a down regulation of defensin 2B at 12 h post parasitism. Similar up-regulations of these AMPs were also observed and reported by Sanda et al. (2018) as part of the early data generated from this project. The expression level of attacin C1, attacin C2, attacin C3, and defensin 2B were highly expressed in *O. nipae* larvae upon infection by *S. carpocapsae* (Sanda et al., 2018). This study therefore aimed to survey the expression pattern of the *O. nipae* signaling gene, Relish in larvae challenged with *H. bacteriophora*, *S. carpocapsae* and their symbiotic bacteria. Similarly, the expression level of AMP families of attacin and defensin were also explored after infection of *O. nipae* larvae with *H. bacteriophora*, *X. nematophila*, and *P. luminescens*. Lastly, we knocked down Relish and ascertained its role on AMPs expressions upon challenge with nematodes and their symbiotic bacteria.

## Materials and Methods

### Insect Rearing

The beetle was reared as previously described by Hou et al. (2014a, b). Briefly, *O. nipae* was collected from Fuqing Entry-Exit Inspection and Quarantine Bureau, Fujian Province, China (25°43′42″N, 138 119°20′35″E) from infested *P. canariensis* Hort. Ex Chabaud Nursery. Individuals of all stages were collected and maintained in our laboratory before use. They were fed on leaves of fortune windmill palm, *Trachycarpus fortunei* (Hook). They were kept in a perforated plastic box (70 mm diameter, 105 mm height) and maintained in a climate chamber at 25 ± 1°C, 80 ± 5% RH, as well as a 12:12 h (light: dark) photoperiod (Ali et al., 2018a).

### Nematodes and Bacterial Cultures

The EPNs used were cultured in our laboratory as reported by Sanda et al. (2018). In summary, *H. bacteriophora* and *S. carpocapsae* were obtained from Guangdong Institute of Applied Biological Resources, China (Yan et al., 2013) and cultured using White trap method (Grewal et al., 1993). The free living stage of the nematode called infective juveniles (IJs), were collected and kept in distilled water at 13°C. Nematodes were allowed to acclimatize to room temperature for at least 30–60 min before use (Georgis and Kaya, 1998).

The symbiotic bacteria *P. luminescens* and *X. nematophila* were isolated from the haemolymph of *Galleria mellonella* infected with IJs of *H. bacteriophora* and *S. carpocapsae*, respectively. Dead *G. mellonella* larvae (2–3 days after inoculation) were surface-sterilized in 70% alcohol for 10 min, flamed and kept in a laminar airflow cabinet for 2 min to dry. Larvae were dissected with sterile needles and scissors, and a needle was used to streak one drop of the oozing haemolymph onto NBTA plates (Fukruksa et al., 2017). The agar plates were sealed and incubated in the dark at 28°C for 48 h. Preliminary identifications of the bacteria were carried out by morphological observation of the colonies. The colonies of the species belonging to the genus *Xenorhabdus* were found to be convex, dark blue, swarm, and umbonated while those of *Photorhabdus* were convex, dark green, and umbonated (Vitta et al., 2017). Each of the colonies was isolated and further cultured on another NBTA media to obtain uniform colonies for further identification. Subsequently, a single colony was selected and cultured on LB with shaking (600 rpm) at 28°C for 20 h. We determined bacterial concentration from the broth by optical density (OD) measurement using a spectrophotometer at 600 nm wavelength (Thanwisai et al., 2012).

### Nematodes and Bacterial Infection Assays

*H. bacteriophora* and *S. carpocapsae* were used to infect the *O. nipae* third instar larvae at 100 IJs per larva in 96-well plates (Dobes et al., 2012; Sanda et al., 2018). The larvae were individually placed in each of the well plates in which 1 × 2 cm tissue papers with small pieces of *T. fortunei* (windmill palm). After 8, 16, and 24 h of treatment, samples were taken from five larvae each for further analyses. For bacterial injection, the bacterial concentrations were determined by adjusting the bacterial suspension to 0.2 at OD_600 nm_ using spectrophotometer for *X. nematophila* and *P. luminescens* prior to injection. 112 nL each of *X. nematophila* and *P. luminescens* suspended in water were injected at concentrations of 2.3 × 10^6^, and 2.9 × 10^6^ CFU/ml, respectively. Distilled water-injected larvae were used as controls. All insects were kept at the climate chamber before use and samples were taken at 8, 16, and 24 h post injection. Thirty individual larvae for each treatment were used and replicated three times to confirm the results.

### RNA Isolation and Complementary DNA (cDNA) Synthesis

Prior to nematodes and bacterial challenges, samples were collected from different tissues of *O. nipae* larvae such as head, fat body, gut, and hemolymph for expression profiles of Relish. Dissections of larvae were carried out in saline buffer solution containing 150 mM NaCl, 2.7 mM KCl, 1.8 mM KH_2_PO_4_, and 10.1 mM Na_2_HPO_4_, pH 7.4, under a stereomicroscope. Each replicate contained more than 40 larvae. Total RNA was extracted from the *O. nipae* larvae (infected and control) using TRIzol reagent (Invitrogen, Carlsbad, CA, United States) as described in the manufacturer’s manual. Integrity and concentration of the isolated RNA were checked by agarose gel electrophoresis and using NanoDrop 2000 analyzer (Thermo Fisher Scientific Inc., Waltham, MA, United States), respectively. It was then used to synthesize cDNAbyqPCR (One-Step gDNA Removal) (TransGen-TransScript, Beijing, China).

### Quantitative Real-Time RT-PCR (qRT-PCR) of Relish and Selected AMPs

Samples of cDNA from the infected larvae were used for qRT-PCR analysis. Primers were synthesized by Fuzhou TSINGKE Biological Technology (Fuzhou, China). For each biological replicate, the reactions were carried out in triplicate in 20 μL reaction volume each containing 1 μL of 500 nM primers, 1 μL of diluted cDNA (diluted 10-fold), 8 μL of sterile water and 10 μL of FastStart universal SYBR Green Master Mix (Roche, Basel, Switzerland), as previously reported by Sanda et al. (2018). All calculations were done following the accompanying ABI 7500 system software and using ribosomal protein S3 (rpS3) as a reference gene. Sequences of the primers are shown in Supplementary Table S1.

### Cloning and Sequence Analysis

TransScript^®^IISuperMix (TransGen-TransScript, Beijing, China) was used to synthesize cDNA. The gene sequence was obtained from the transcriptome database of *O. nipae* (Tang et al., 2014a). To confirm the sequences obtained by deep sequencing, we designed primers (Supplementary Table S1) using Primer Premier 5.0 software and synthesized at Fuzhou TSINGKE Biological Technology (Fuzhou, China). Polymerase chain reaction (PCR) was performed using the Trans2x EasyTaq^®^ PCR SuperMix (TransGen-TransScript, Beijing, China) using the following parameters: an initial delay at 94°C for 5 min; 30 cycles of denaturation at 94°C for 30 s, annealing at 55°C for 30 s, and extending at 72°C for 1 min; and a final extension at 72°C for 5 min. The generated PCR products were then purified using EasyPure^®^ Quick Gel Extraction Kit (TransGen-TransScript, Beijing, China) and finally cloned into the pEASY^®^ T1 Vector (TransGen-TransScript, Beijing, China) in accordance with the manufacturer’s instructions. The positive colonies were picked and sent to Sanger sequencing at TsingKe Biological Technology (Guangzhou, China). The partial sequence was analyzed with the NCBI BLAST tool^1^ and SMART^2^ to identify its ORF and predict its functional domains, respectively. The multiple sequence alignments and phylogenetic analysis were conducted using MEGA 5.05^3^.

### The RNA Interference (RNAi) and dsRNA Injection

For silencing of *O. nipae* Relish, we synthesized double-stranded RNA (dsRNA) using a MEGAscript^®^ RNAi kit (Thermo Fisher Scientific) following the manufacturer’s protocols. Primers (Supplementary Table S1) which contained the T7 promoter region in both sense and antisense strands were designed at https://www.dkfz.de/signaling/e-rnai3/idseq.php E-RNAi and synthesized at Fuzhou TSINGKE Biological Technology (Fuzhou, China). The concentration of the dsRNA was checked using a NanoDrop 2000 where it was further adjusted to 750 ng/μL. A 110 ng of Relish dsRNA was injected to each larva. Infected larvae were transferred to new petri dishes and fed with small *T. fortunei* leaves. Five larvae were used to extract total RNA at 16 and 24 h after injection for expression analysis as described above. Negative control using dsEGFP was compared with the treatments.

### Expressions of Selected Antimicrobial Peptides (AMPs) After Relish Knock Down and Nematode-Bacterial Infection

To check the effects of microbial challenge on the expression levels of some selected AMPs after OnRelish knock down, larvae were infected with *S. carpocapsae* and *H. bacteriophora* at 16 h post Relish dsRNA injection at 100 IJs per larva. Infected larvae were transferred to new petri dishes provided with small *T. fortunei* leaves as feed. Similarly, *X. nematophila* and *P. luminescens* were injected at 16 h post Relish dsRNA injection. Total RNA was extracted from five larvae and used for qPCR analyses of AMPs expressions as described above. Negative control with dsEGFP was compared with the treatments.

### Data Analyses

The mean expression levels of the treatments at three different time-points were compared and analyzed using One-way analysis of variance (ANOVA). This was then followed by Duncan’s multiple range test (DMRT) at 95% confidence level (*P* 0.05). This method was also applied to the control treatment values at three different time-points. Expression levels of AMPs and Relish were transformed by logarithmic function where necessary. Gene expression data between the infected and control treatments at a particular time-point were analyzed using Student’s *t*-test. Differences between mean values were analyzed and considered significant when *P* < 0.05 or considered extremely significant when *P* < 0.0001 concerning the control values. All analyses were performed using IBM SPSS Statistics version 22 (IBM Corporation, New York, NY, United States) (SPSS, RRID: SCR_002865).

## Results

### *X. nematophila* and *P. luminescens* and Their Symbiotic Nematodes Induce Strong Up-Regulation of Relish at 24 h Post Infection

**FIGURE 1:**
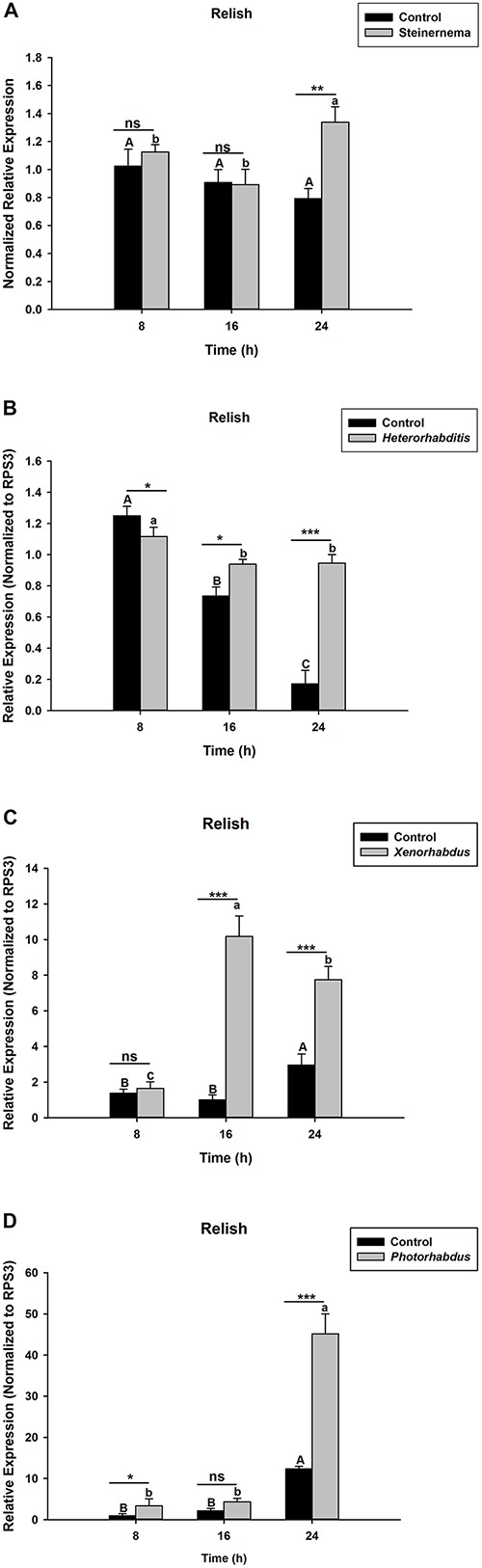
Transcription levels of Relish in *O. nipae* larvae infected with; **(A)** *S. carpocapsae* **(B)** *H. bacteriophora* **(C)** *X. nematophila* **(D)** *P. luminescens*. Error bars labeled with different letters are significantly different (one-way ANOVA followed by LSD test, *p* < 0.05). ^∗∗∗^*P* < 0.0001; ^∗∗^*P* < 0.001; ^∗^*P* < 0.01 indicates significantly different levels between the control and pathogen treatments at the indicated time period; while “ns” indicates no significant difference.

The activation of *Relish*, a NF-κB transcription factor, is known to be triggered by IMD pathway upon microbial challenges to induce the expression of AMP genes. For this reason, we assessed the expression level of OnRelish upon the EPN-bacteria complex infections. The results indicate that, upon *S. carpocapsae* and *H. bacteriophora* infections, the expression levels of OnRelish were up-regulated at 24 h after treatments (*t*_4_ = 2.17, *P* = 0.002)(Figures 1A,B). However, in both treatments, similar expression levels of the gene (with respect to the control) were observed at early hours (8 and 16 h) of infection. On the other hand, injections of the symbiotic bacteria *X. nematophila* and *P. luminescens* also affected the OnRelish gene expression at certain time-points. At 8 h post injection, no difference was observed in the gene expression with respect to *X. nematophila* infection (Figure 1C); but a weak up regulation was observed at this time-point when the host was infected with *P. luminescens* (Figure 1D). At 16 hours post infection (hpi), *X. nematophila* induced a strong up-regulation of OnRelish while no difference observed in the case of *P. luminescens* infection. Both symbiotic bacteria caused strong up regulation (*t*_4_ = 1.93, *P* = 0.001) of the gene with respect to the control at the late hour (24 hpi) (Figures 1C,D). We therefore conclude that *S. carpocapsae* and *H. bacteriophora* and their respective symbiotic bacteria induce strong up regulation of Relish in *O. nipae*.

### *X. nematophila* and *P. luminescens* Trigger Up-Regulations of Some AMPs at Nearly All Time-Points

**FIGURE 2:**
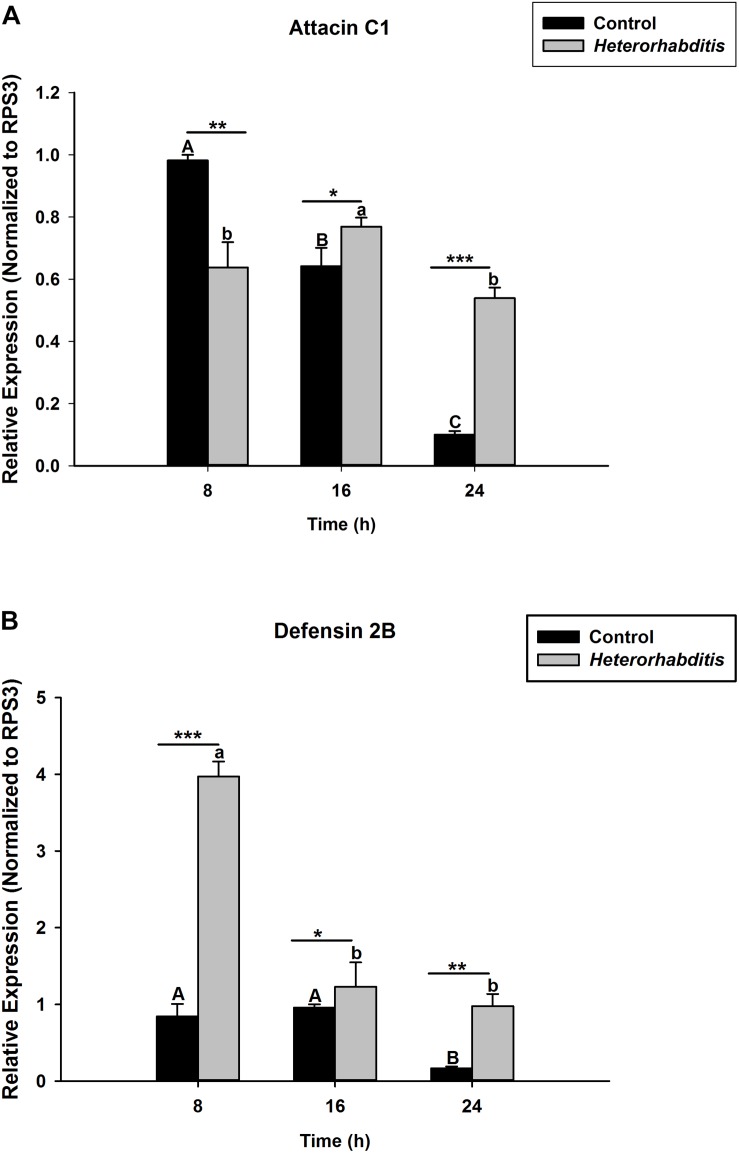
Transcription of antimicrobial peptide (AMP) genes in *O. nipae* larvae infected with *H*. *bacteriophora* AMP transcription levels were shown for **(A)** Attacin C1, **(B)** Defensin 2B. Error bars labeled with different letters are significantly different (one-way ANOVA followed by LSD test, *p* < 0.05). ^∗∗∗^*P* < 0.0001; ^∗∗^*P* < 0.001; ^∗^*P* < 0.01) indicates significantly different levels between the control and *H*. *bacteriophora* treatments at the indicated time period; while “ns” indicates no significant difference.

**FIGURE 3:**
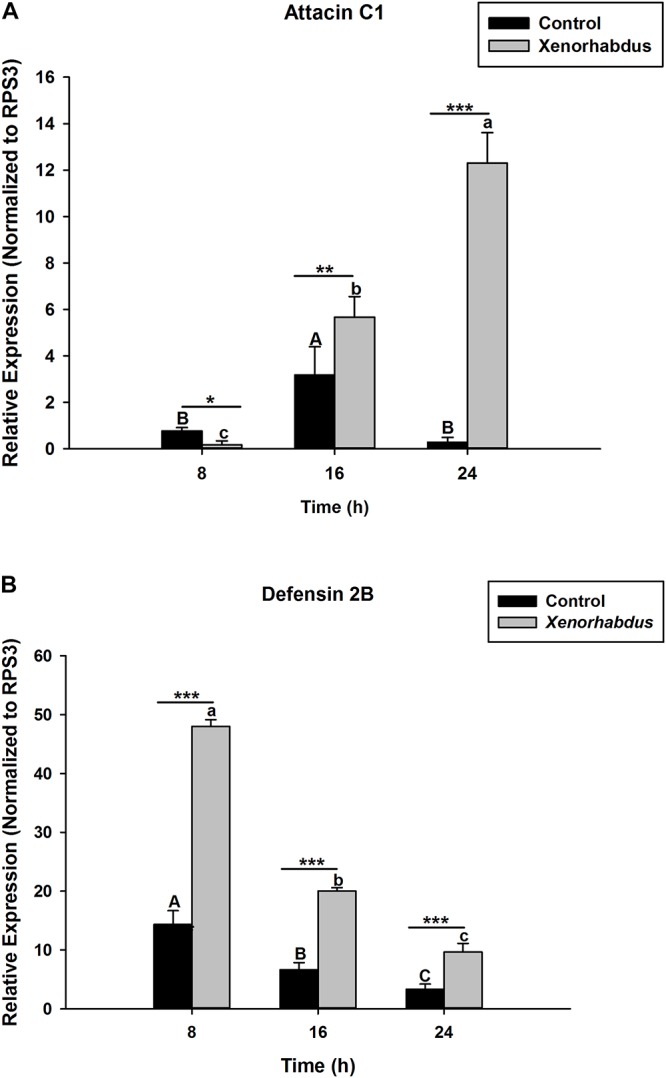
Transcription of antimicrobial peptide (AMP) genes in *O. nipae* larvae injected with *X*. *nematophila* AMP transcription levels were shown for **(A)** Attacin C1, **(B)** Defensin 2B. Error bars labeled with different letters are significantly different (one-way ANOVA followed by LSD test, *p* < 0.05). ^∗∗∗^*P* < 0.0001; ^∗∗^*P* < 0.001; ^∗^*P* < 0.01 indicates significantly different levels between the control and *X*. *nematophila* treatments at the indicated time period; while “ns” indicates no significant difference.

**FIGURE 4:**
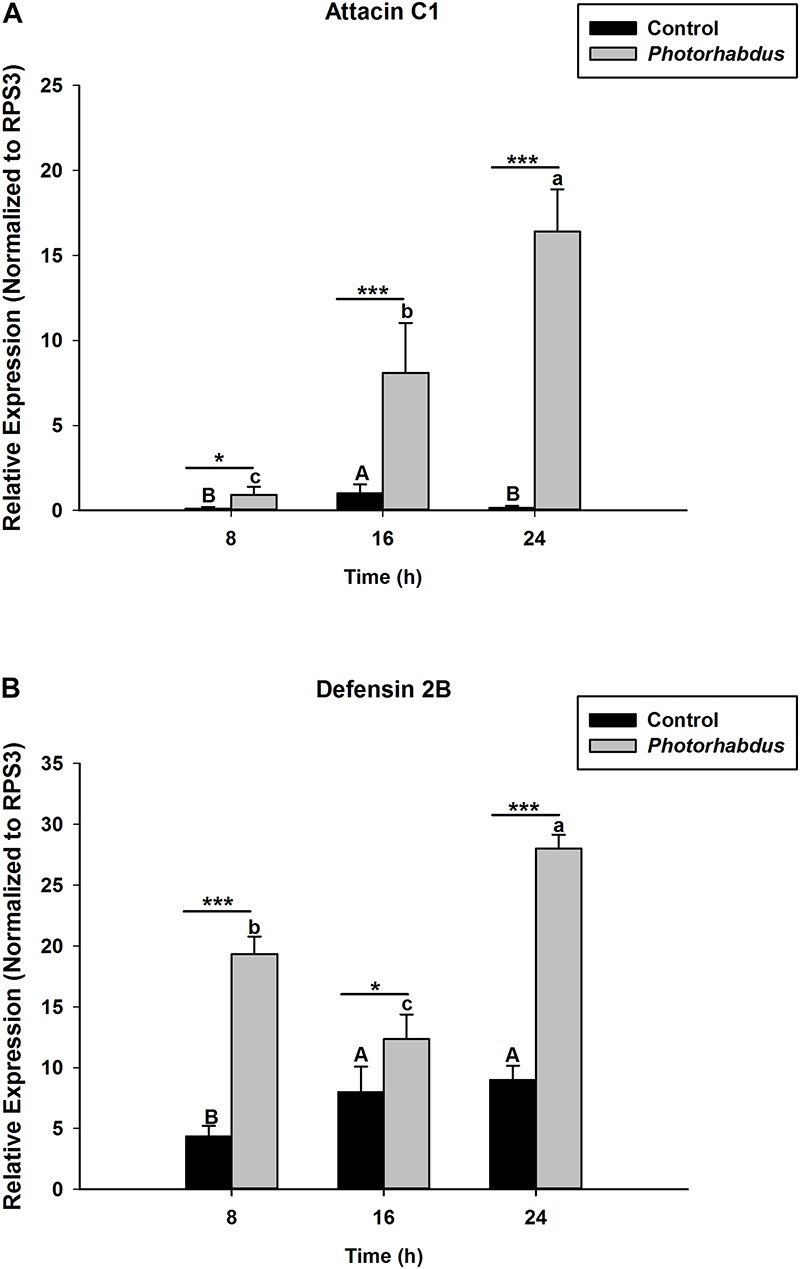
Transcription of antimicrobial peptide (AMP) genes in *O. nipae* larvae injected with *P*. *luminescens* AMP transcription levels were shown for **(A)** Attacin C1, **(B)** Defensin 2B. Error bars labeled with different letters are significantly different (one-way ANOVA followed by LSD test, *p* < 0.05). ^∗∗∗^*P* < 0.0001; ^∗∗^*P* < 0.001; ^∗^*P* < 0.01 indicates significantly different levels between the control and *P*. *luminescens* treatments at the indicated time period; while “ns” indicates no significant difference.

*S. carpocapsae* induces up-regulations of two defensin and three attacin genes in *O. nipae* from our previous experiments (Sanda et al., 2018). Similar results were also obtained here when *O. nipae* were infected with *H. bacteriophora*. There were significant up-regulations of attacin C1, attacin C2 and defensin 2B at different time-points (*t*_4_ = 1.53, *P* = 0.001) (Figures 2A,B and Supplementary Figure S1). Attacin C1 was significantly down-regulated at 8 h after *H. bacteriophora* infections (*t*_4_ = 1.72, *P* = 0.010) (Figure 2A). Similar down regulations of defensin 2A were recorded at both 8 (*P* = 0.001 and *t*_4_ = 2.07) and 16 h (*P* = 0.001 and *t*_4_ = 1.31) after infection, except at 24 h (Supplementary Figure S2) where there was significant up-regulation of defensin 2A at 24 h after *H. bacteriophora* (*t*_4_ = 1.74, *P* = 0.032). We went further to test the expression levels of these AMPs after symbiotic bacterial injections. We found out that the pattern of mRNA expression levels of all the AMPs induced by *X. nematophila* and *P. luminescens* were up-regulated at all time-points (Figures 3A,B, 4A,B and Supplementary Figure S3) except where down regulation was observed for attacin C1 at 8 hpi (Figure 3A) and defensin 2A at 8 and 16 hpi (Supplementary Figure S4), all due to *X. nematophila* infection. Other exceptions in relation to *X. nematophila* infection could be seen in Supplementary Figures S5, S6).

### Transcriptional Profiles of Relish in the Absence of Microbial Infections Across Different Tissues in *O. nipae* Larvae

**FIGURE 5:**
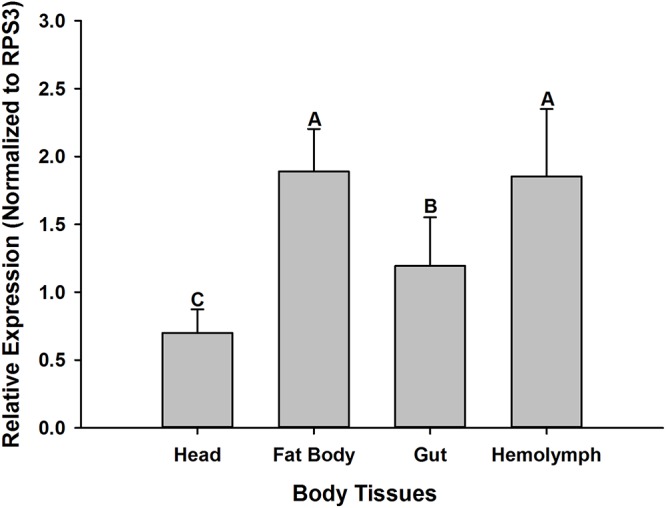
Expression profiles of *OnRelish* across different tissues in the absence of microbial challenge. The data were presented as Mean ± SD (*n* = 3). Statistical significance is indicated by different letters above the bar at *P* < 0.001.

In the absence of microbial infections, the RT-qPCR data reveals that OnRelish mRNA was expressed in all the tissues assayed for, including the head, fat body, gut and hemolymph. Significant differences were detected in the abundance of OnRelish transcript across different tissues (ANOVA: *F*_2_,_6_ = 13.037, *P* < 0.05). The highest transcript levels were found in the fat body and hemolymph (Figure 5). However, there were non-significant differences between the fat body and hemolymph in terms of OnRelish transcription level.

### Molecular Confirmation of Relish in *O. nipae* Larvae and Phylogenetic Analysis

**FIGURE 6:**
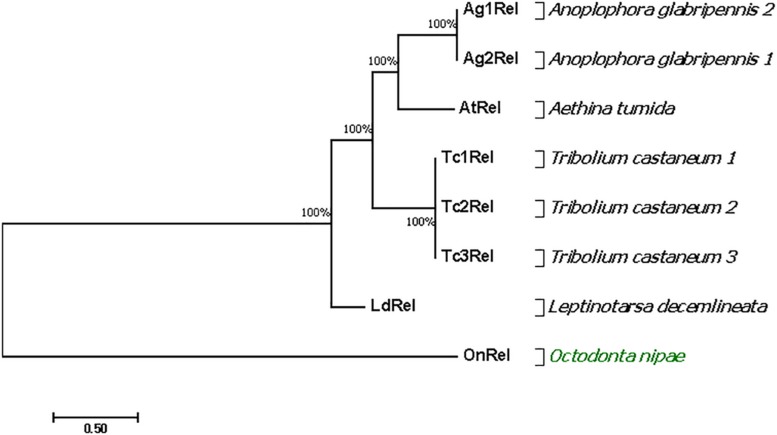
Phylogenetic relationships of *O. nipae* Relish and other arthropod Relish. The amino acid sequences of the partial OnRelish proteins and complete proteins of other arthropod NF-κB p110/Relish were aligned to construct the maximum likelihood (ML) tree using MEGA 7.0. Numbers indicate the percentage of bootstrap replications that support each branch.

The Relish gene fragment was obtained from the *O. nipae* transcriptome database. The cDNA is 571 bp with an ORF of 102 bp. The *O. nipae Relish* encodes a predicted polypeptide of 102 amino acid residues and includes an amino-terminal SCOPd1gnla (3–44), DEATH domain (7–96) (proteins involved in apoptosis), Pfam: DUF4888 domain (4–72), GatB_Yqey (8–99) and transmission membrane regions at 68–90. The structural characteristics above show that the encoded protein is a Relish/NF-κB p110 subunit-like homolog named here as *OnRelish*. Further, our search of non-redundant protein sequences from the NCBI database indicated that the *OnRelish*/NF-κ-B p110 subunit-like proteins were most similar to *Leptinotarsa decemlineata* (XM_023174540.1), *Anoplophora glabripennis* 1 (XM_018710311.2), *A. glabripennis* 2 (XM_018710307.1), *Tribolium castaneum* 1 (XM_008196263.2), *T. castaneum* 2 (XM_965801.3), *T. castaneum* 3 (XM_965801.3) and *Aethina tumida* 1 (XM_020011061.1) (LR134433.1) with 51, 46, 46, 43, 43, 43, and 42% identity in amino acid sequences, respectively (Supplementary Figure S7). To illustrate the evolutionary relationship between OnRelish/NF-κ-B and other arthropod Relish/NF-κ-Bs, we constructed a Maximum Likelihood tree through alignment of 8 Relish protein sequences obtained from *L. decemlineata*, *A. glabripennis* 1, *A. glabripennis* 2, *T. castaneum* 1, *T. castaneum* 2, *T. castaneum* 3, and *A. tumida* 1 (Figure 6). *OnRelish* clustered closely with some Relish proteins from other insects including *L. decemlineata*, suggesting a close ancestral origin, and *OnRelish* was considered an ortholog of *L. decemlineata* Relish. Taken together, these results suggest that *OnRelish* has a close evolutionary relationship with Relish protein from these species.

### Silencing Efficiency of OnRelish dsRNA

**FIGURE 7:**
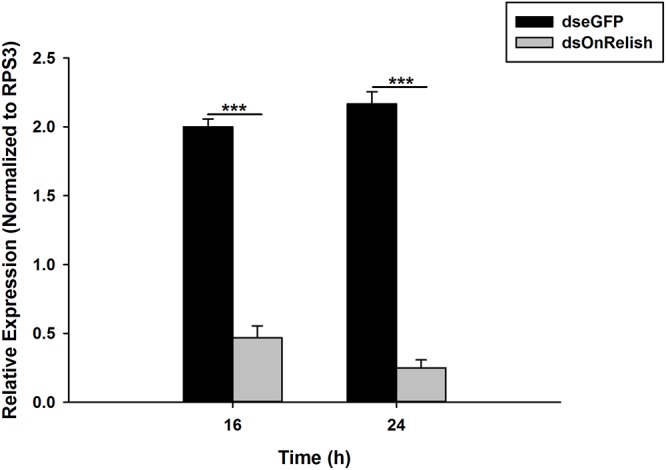
Detections of the RNAi efficiency of OnRelish. Relative mRNA expression levels of OnRelish by qRT–PCR. dsOnRel and dsGFP were injected to third instar larvae and samples were taken at 16 and 24 h after injection. ^∗∗∗^*P* < 0.0001 indicates significantly different levels between two treatments.

Silencing of OnRelish gene was achieved through RNAi by injection of dsRNA and verified by qPCR analysis. The results revealed a significant decrease in the OnRelish mRNA transcript levels with RNAi efficiencies of 77% at 16 h (*t*_4_ = 14.54, *P* = 0.00) and up to 88% at 24 h (*t*_4_ = 18.13, *P* = 0.001), compared with those of EGFP dsRNA injection (Figure 7).

### Expression Profiles of AMPs After OnRelish Knock Down and Microbial Challenges

**FIGURE 8:**
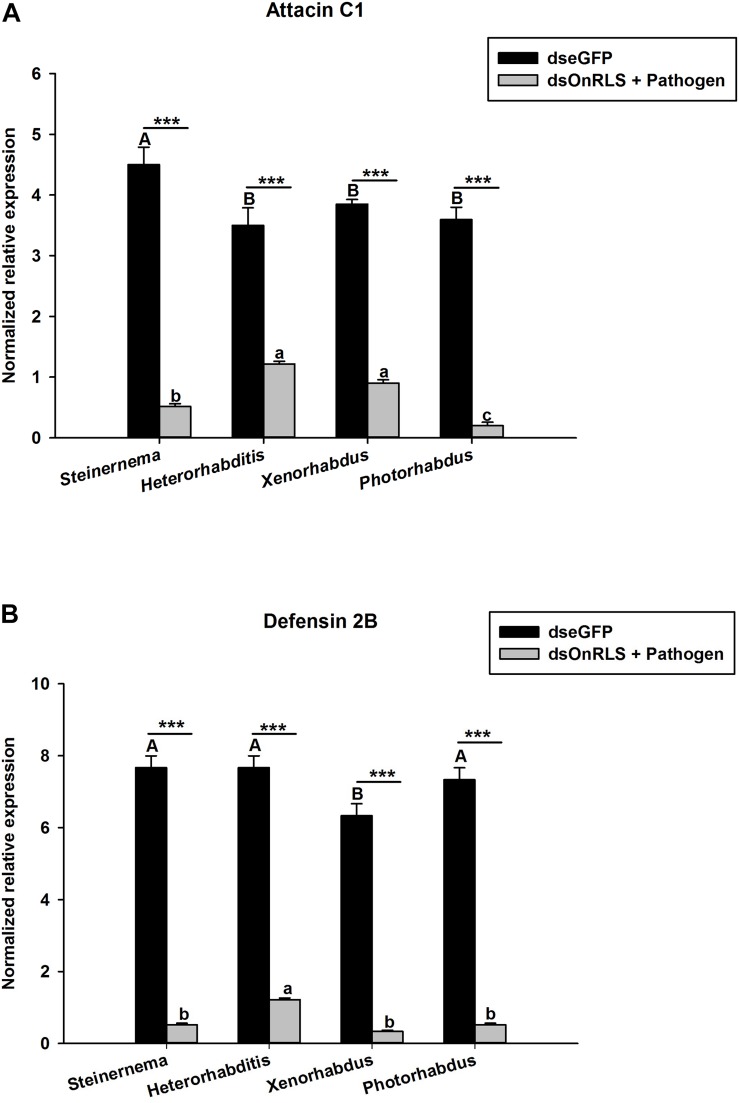
Effects of OnRelish gene silencing on the transcription level of antimicrobial peptide gene; **(A)** Attacin C1 and **(B)** Defensin 2B in larvae of *O*. *nipae* after Microbial challenge. S.c. – *S. carpocapsae*, H.b. – *H*. *bacteriophora*, *X. nematophila* and *P. luminescens*. Error bars labeled with different letters are significantly different (one-way ANOVA followed by LSD test, *p* < 0.05). ^∗∗∗^*P* < 0.0001 indicates significantly different levels between the control and microbial treatments at the indicated time period; while “ns” indicates no significant difference.

To investigate whether the silencing of OnRelish can affect the expressions of some selected AMPs in *O. nipae* larvae, the transcription levels of Attacin C1 and Defensin 2B after the EPNs and their symbiotic bacterial challenges were investigated. The results show that, at 24 h post-treatments, the transcription levels of Attacin C1 and Defensin 2B in dsOnRelish plus pathogen-injected groups appeared significantly lower than those of the various control groups (Figures 8A,B). In Figure 8A, Attacin C1 was significantly down-regulated in *S. carpocapsae* (*t*_4_ = 3.1, *P* = 0.00), *H. bacteriophora* (*t*_4_ = 2.8, *P* = 0.001), *X. nematophila* (*t*_4_ = 4.31, *P* = 0.001) and *P. luminescens* (*t*_4_ = 3.876, *P* = 0.001) treated larvae. Similarly, Defensin 2B were significantly down-regulated across the four treatments compared to control (*t*_4_ = 2.6, *P* = 0.001) (Figure 8B). Therefore, the results provide evidence that OnRelish played an important role in mediating the immune expressions of AMPs in *O. nipae* larvae against microbial infections.

## Discussion

Cellular and humoral responses are integral parts of innate immune defenses which appear to be conserved in animals. These immune mechanisms are regulated by signaling pathways due to stimuli including (but not limited to) pathogen recognition by immune systems upon microbial invasions (Marmaras and Lampropoulou, 2009). Among the insect’s immune responses to the pathogenic invasion, AMPs are the last line of defense in insect, unlike cellular immune process that is immediate. However, the expressions of these AMPs are believed to be induced by rapid proteolytic cleavage of the transcription factor Relish. Relish is a member of Rel/NF-κB family, important for humoral immune reaction in *Drosophila* (Dushay et al., 1996). According to Hedengren et al. (1999) and Leulier et al. (2000), Relish regulate the immune effector genes encoding cecropin, diptericin, attacin, metchnikowin and defensin in *D. melanogaster*.

Similarly, in *Drosophila*, EPNs infections lead to activation of IMD pathway, which induces the activation of some AMP genes. Infecting *Drosophila* with *Heterorhabditis-Photorhabdus* complex induces the expression of attacin genes as well as diptericin, metchnikowin and drosomycin at 24 h post-treatments (Peña et al., 2015). In this study, our results indicated that the expression levels of OnRelish were up-regulated upon *S. carpocapsae* and *H. bacteriophora* infections as well as those of their symbiotic bacteria especially at 24 h after treatments. However, we observed some differences in the expression levels of the controls at 24 hpi when comparing (Figures 1A,B) despite similar treatments. It is probable that this might be due to different cell cycle stages of the two independent samples as at the time of RNA isolation.

The hypothesis that *Relish* expression is triggered from IMD pathway upon microbial challenges to induce AMP genes expressions holds true in our study. The expression of OnRelish upon the nematodes-bacteria complex also resulted in the positive regulations of two AMPs gene families in *O. nipae*. There were significant up-regulations of attacin C1, attacin C2, and defensin 2B at 8, 16, and 24 h after *H. bacteriophora* infections. Similar up-regulations of these AMPs in *X. nematophila* and *P. luminescens* were also reported. Thus, among the nematodes-bacteria complex challenges, *P. luminescens* induces higher up-regulations of AMPs in *O. nipae*. A previous study by Sanda et al. (2018) in *O. nipae* reported that *S. carpocapsae* induces the up-regulations of attacin C1, attacin C2, and defensin 2B at 8, 16, and 24 h after treatments. Yadav et al. (2017) reported an up-regulation of attacin-A, B, C, and drosomycin each at 6 and 24 h post *S. carpocapsae* infections.

However, the pattern of AMP expressions in some insects is irregular and varies with the type of microbial challenge. This is seen in the study of Darsouei et al. (2017) where cecropin, attacin and spodoptericin expression levels in *H. bacteriophora*-treated larvae were less than those of *S. carpocapsae*-treated ones. This can be linked to the hypothesis that the nematodes and their symbiotic bacteria produce certain toxins to suppress the insect’s immune responses, including the expression of AMPs. This is supported by Castillo et al. (2015) where *Photorhabdus* produces several toxins and virulence factors that suppress major immune mechanisms of their hosts including degradation of AMPs. Similarly, Hwang et al. (2013) reported that *X. nematophila* inhibits insect AMP gene expressions by shutting down the eicosanoid signaling. Shrestha and Kim (2009) also reported that in addition to inhibition of AMPs gene expressions, *X. nematophila* can suppress humoral immune responses. We therefore joined previous authors to raise a question of how these nematodes and their symbiotic bacteria inhibit the expression of AMP genes? Overall, our data is thus similar to the ones reported in Dipterans particularly in *Drosophila*, where most AMPs are positively expressed in response to nematodes-bacteria complex infections (Aymeric et al., 2010; Darsouei et al., 2017; Yadav et al., 2017). Therefore, we hold the opinion that the stages of immunity suppressed by these pathogens are absent in *Drosophila* with regards to cellular and humeral responses in general (Aymeric et al., 2010; Casanova-Torres and Goodrich-Blair, 2013), and as well as with regards to AMPs expressions in *O. nipae* in particular.

Furthermore, analysis of the transcription profiles of OnRelish across different tissues of *O. nipae* in the absence of microbial infections showed that the gene is expressed in all tested tissues including head, fat body, gut and hemolymph. However, the highest expression of Relish was detected in the fat body and hemolymph. Imler and Bulet (2005) also reported higher transcription of AMPs in the fat body and hemocytes. Similar to human liver, fat body is a major immune organ which can produce AMPs in additions to vast arrays of immune molecules (Lemaitre and Hoffmann, 2007). Similarly, many different AMPs are secreted into the hemolymph of insects to combat and rapidly respond to various invasion attempts by pathogens (Sun et al., 2013). Thus, the high transcription level of OnRelish in hemolymph and fat body might suggest that OnRelish is involved in the activation and regulation of immune defenses in *O. nipae*.

A DEATH domain (proteins involved in apoptosis) was detected at 7–96 bp in the amino acid sequence of OnRelish. Relish belongs to the super family of Death Domain-(DD) containing proteins with striking similarity to mammalian p105 DD (Beinke et al., 2002; Myllymaki et al., 2014). The p110 is a catalytic subunit of PI 3-kinase that is required for the activation of NF-κB-dependent reporter genes. Cell death is mediated by NF-κB homolog of Relish at its C-terminus in *Drosophila* (Georgel et al., 2001). Other domains found present in OnRelish include an amino-terminal SCOPd1gnla (3–44), DEATH domain (7–96) (proteins involved in apoptosis), Pfam: DUF4888 domain (4–72), GatB_Yqey (8–99) and transmission membrane regions at 68–90. Phylogenetic analysis of the partial sequences showed that OnRelish *O. nipae* clustered with registered Relish/NF-κB p110 proteins of other insect species especially *L. decemlineata* which came from the same order (Coleoptera) and family (Chrysomelidae) with *O. nipae*. We, therefore, conclude that the OnRelish cloned sequence belongs to the Rel/NF-κB family.

To further clarify the role of OnRelish in the immune response to nematodes and symbiotic bacterial infection, the effect of dsRNA-mediated gene silencing on the transcription of the genes for the antimicrobial peptides attacin C1 and defensin 2B was tested. The results indicated that the activities of the two peptides in the *S. carpocapsae* and *H. bacteriophora* infected larvae were significantly inhibited after OnRelish was knocked down. Similar, results were also obtained after *X. nematophila* and *P. luminescens* injections. This confirmed the role of OnRelish as the regulator of AMP genes for immune defense in *O. nipae*. The study of Leulier et al. (2000) also confirmed the role of Relish as a regulator of Cecropin, Diptericin, Attacin, Metchnikowin, and Defensin in *D. melanogaster*. Taken together, Relish is important for the activation and regulation of insect immune defense against EPNs and their symbiotic bacteria (Tzou et al., 2002).

## Conclusion

Here we investigated the role of the IMD signaling gene Relish in the regulation of AMPs upon nematodes and symbiotic bacterial infections, before and after gene knock down in *O. nipae*. We found out that the two *O. nipae*’*s* AMP gene families were up-regulated at most of the time-points used, following infection with *S. carpocapsae*, *H. bacteriophora*, *X. nematophila*, and *P. luminescens*. However, knock down of OnRelish resulted in the down regulation of the selected AMPs in both treatment groups compared to the control. Thus, regulation of AMP gene expression by the NF-κB factor Relish in *O. nipae* is hereby established, and the signaling pathways which lead to their induction upon detection of pathogens appear to be conserved in insects. Particularly, our study reveals the role of Relish in *O. nipae’*s defense against invading pathogens through AMP expressions. Future investigation is required to describe the full length sequence of Relish in *O. nipae* and the specific mechanisms of *O. nipae* counteraction involving different AMPs.

## Data Availability Statement

The raw data supporting the conclusions of this manuscript will be made available by the authors, without undue reservation, to any qualified researcher.

## Author Contributions

NS and BH conducted sampling and performed the experiments. All authors analyzed the data and wrote the manuscript. YH supervised and critically reviewed the manuscript.

## Conflict of Interest

The authors declare that the research was conducted in the absence of any commercial or financial relationships that could be construed as a potential conflict of interest.

## Footnotes

1 https://blast.ncbi.nlm.nih.gov/Blast.cgi

2 http://smart.embl-heidelberg.de/smart/set_mode.cgi

3 http://www.megasoftware.net
