# Supplementary material for: Exploring the Role of Relish on Antimicrobial Peptide Expressions (AMPs) Upon Nematode-Bacteria Complex Challenge in the Nipa Palm Hispid Beetle, Octodonta nipae Maulik (Coleoptera: Chrysomelidae)
Source: Front Microbiol. 2019 Oct 31;10:2466. doi: 10.3389/fmicb.2019.02466 (PMC6834688; doi:10.3389/fmicb.2019.02466)
Supplement: Supplementary file 1 [file Data_Sheet_1.pdf]

Supplementary Materials

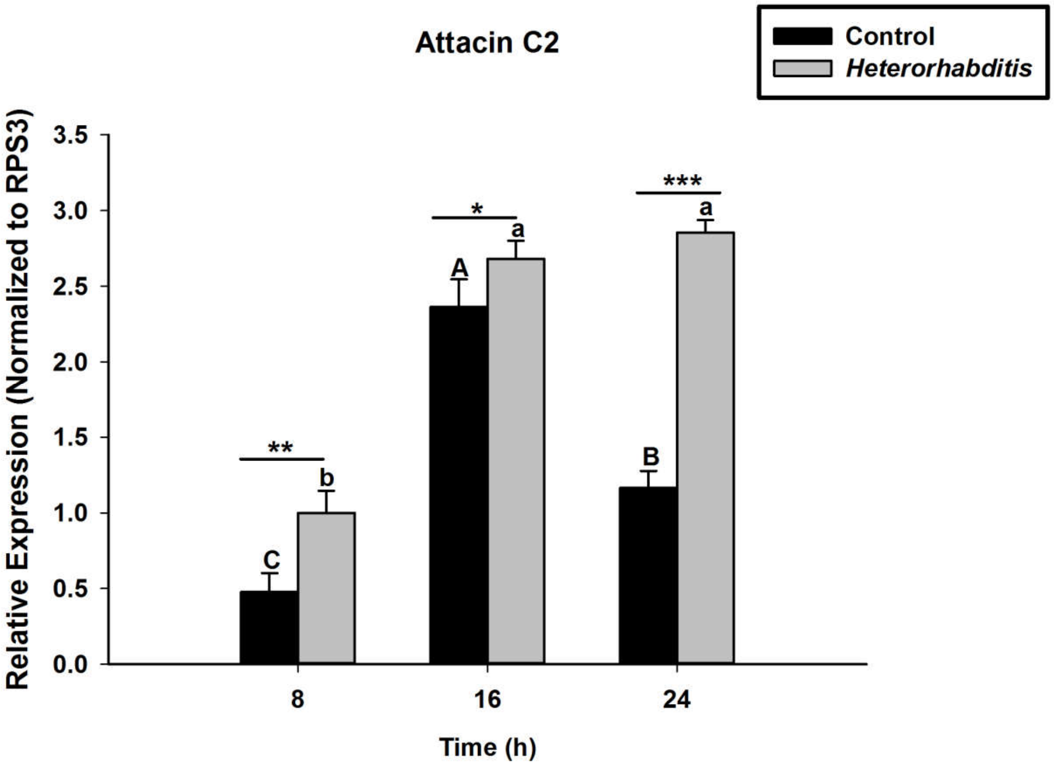

Figure S1: Transcription of antimicrobial peptide (AMP) genes in *O. nipae* larvae infected with *H. bacteriophora* AMP transcription levels are shown for **Attacin C2**. Error bars labeled with different letters are significantly different (one-way ANOVA followed by LSD test, $p < 0.05$). The asterisks *** ($P < 0.0001$); ** ($P < 0.001$); * ($P < 0.01$) indicates different significant levels between the control and H. bacteriophora treatments at the indicated time period; while "ns" indicates no significant difference.

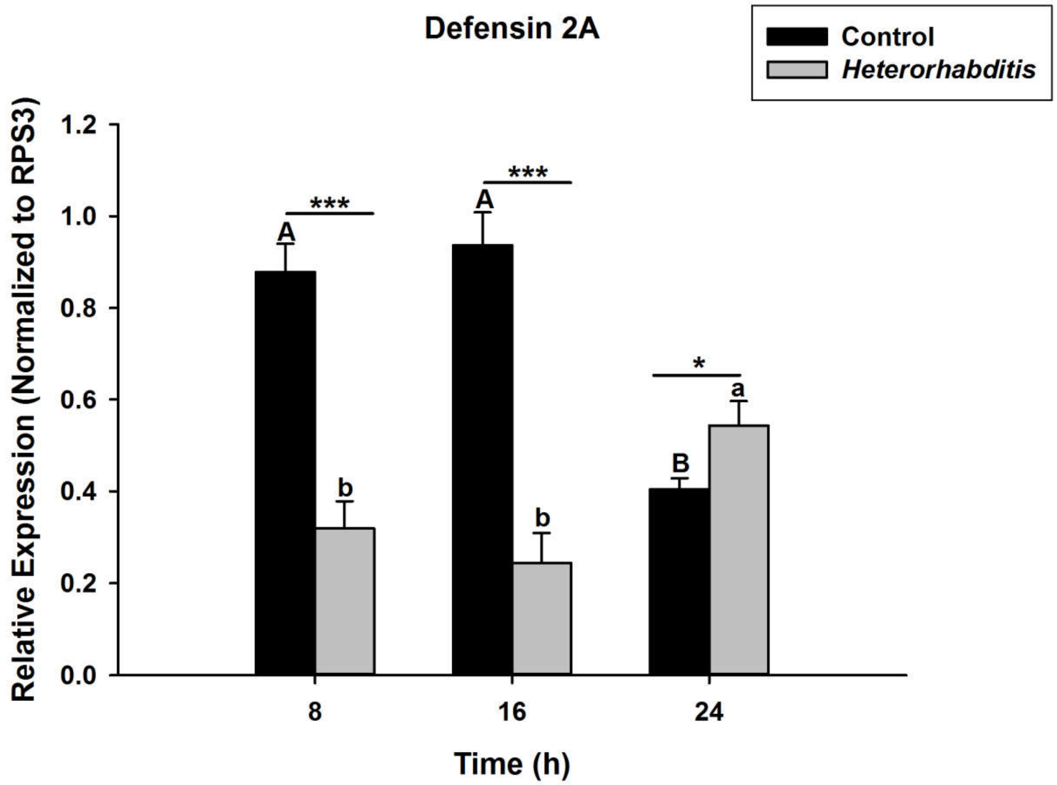

Figure S2: Transcription of antimicrobial peptide (AMP) genes in *O. nipae* larvae infected with *X. nematophila* AMP transcription levels are shown for **Defensin 2 A**. Error bars labeled with different letters are significantly different (one-way ANOVA followed by LSD test, $p < 0.05$). The asterisks *** ($P < 0.0001$); ** ($P < 0.001$); * ($P < 0.01$) indicates different significant levels between the control and *H. bacteriophora* treatments at the indicated time period; while “ns” indicates no significant difference.

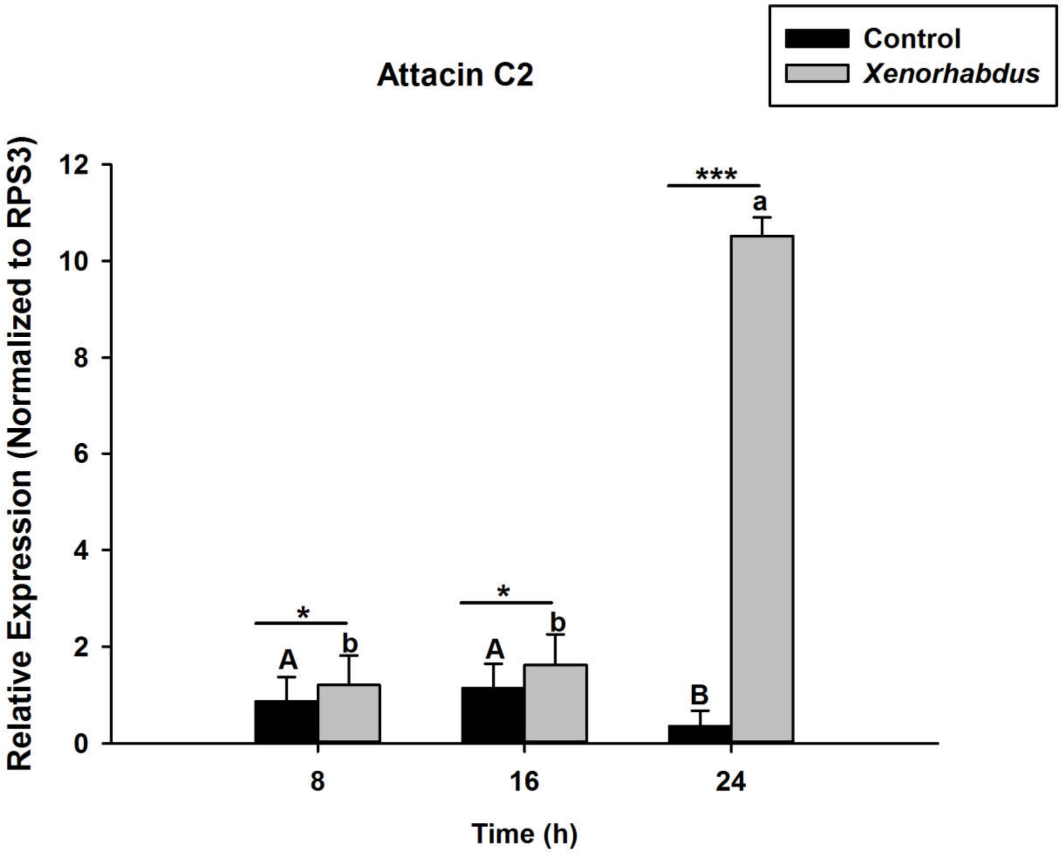

Figure S3: Transcription of antimicrobial peptide (AMP) genes in *O. nipae* larvae infected with *X. nematophila* AMP transcription levels are shown for **Attacin C2**. Error bars labeled with different letters are significantly different (one-way ANOVA followed by LSD test, $p < 0.05$). The asterisks *** ($P < 0.0001$); ** ($P < 0.001$); * ($P < 0.01$) indicates different significant levels between the control and *X. nematophila* treatments at the indicated time period; while “ns” indicates no significant difference.

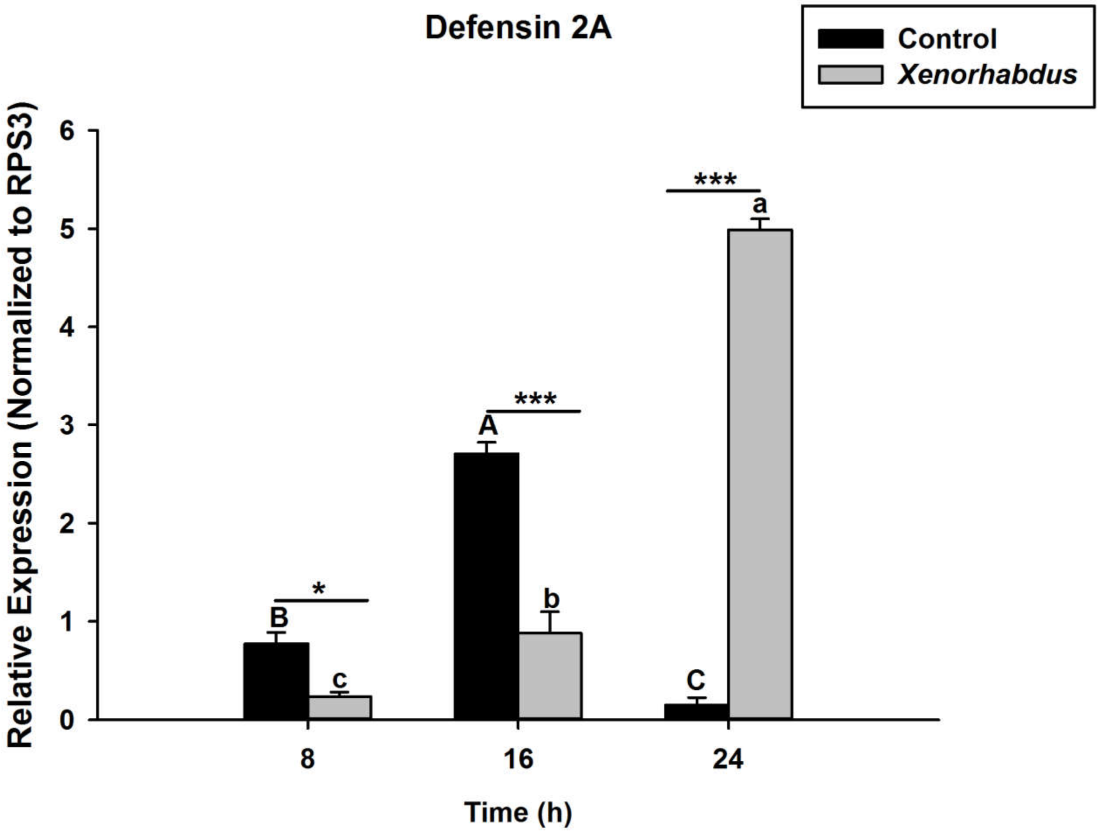

Figure S4: Transcription of antimicrobial peptide (AMP) genes in *O. nipae* larvae infected with *X. nematophila* AMP transcription levels are shown for **Defensin 2A**. Error bars labeled with different letters are significantly different (one-way ANOVA followed by LSD test, $p < 0.05$). The asterisks *** ($P < 0.0001$); ** ($P < 0.001$); * ($P < 0.01$) indicates different significant levels between the control and *X. nematophila* treatments at the indicated time period; while "ns" indicates no significant difference.

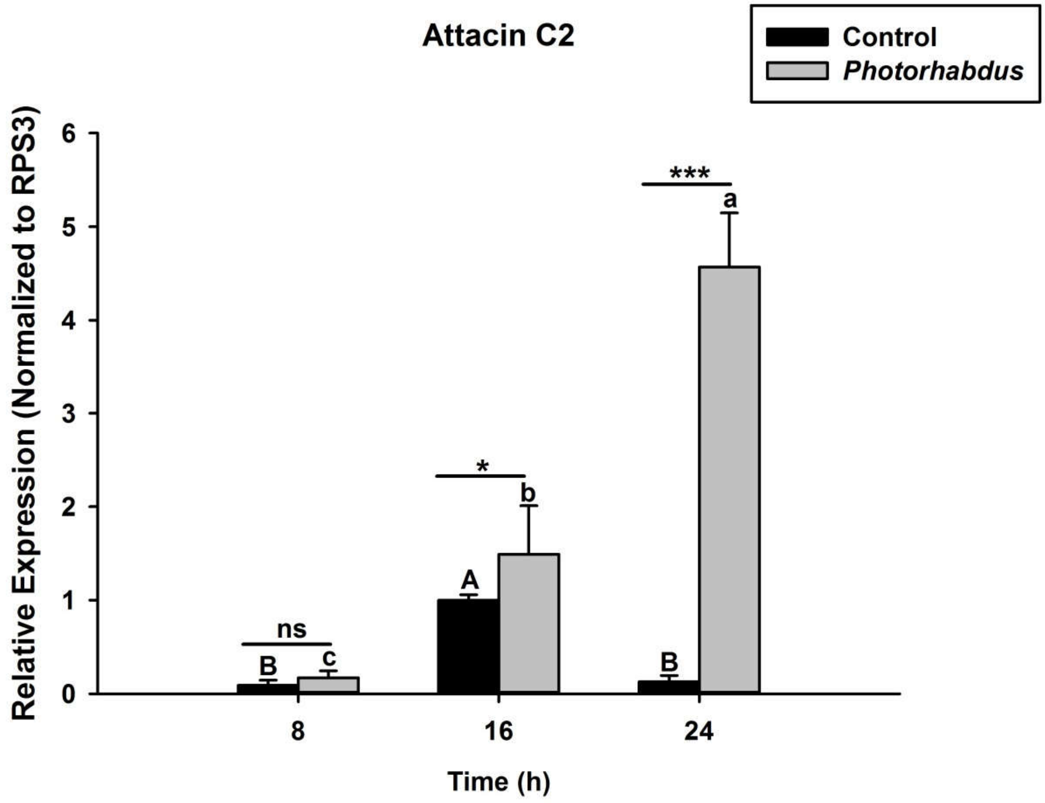

Figure S5: Transcription of antimicrobial peptide (AMP) genes in *O. nipae* larvae infected with *P. luminescens* AMP transcription levels are shown for **Attacin C2**. Error bars labeled with different letters are significantly different (one-way ANOVA followed by LSD test, $p < 0.05$). The asterisks *** ($P < 0.0001$); ** ($P < 0.001$); * ($P < 0.01$) indicates different significant levels between the control and *P. luminescens* treatments at the indicated time period; while "ns" indicates no significant difference.

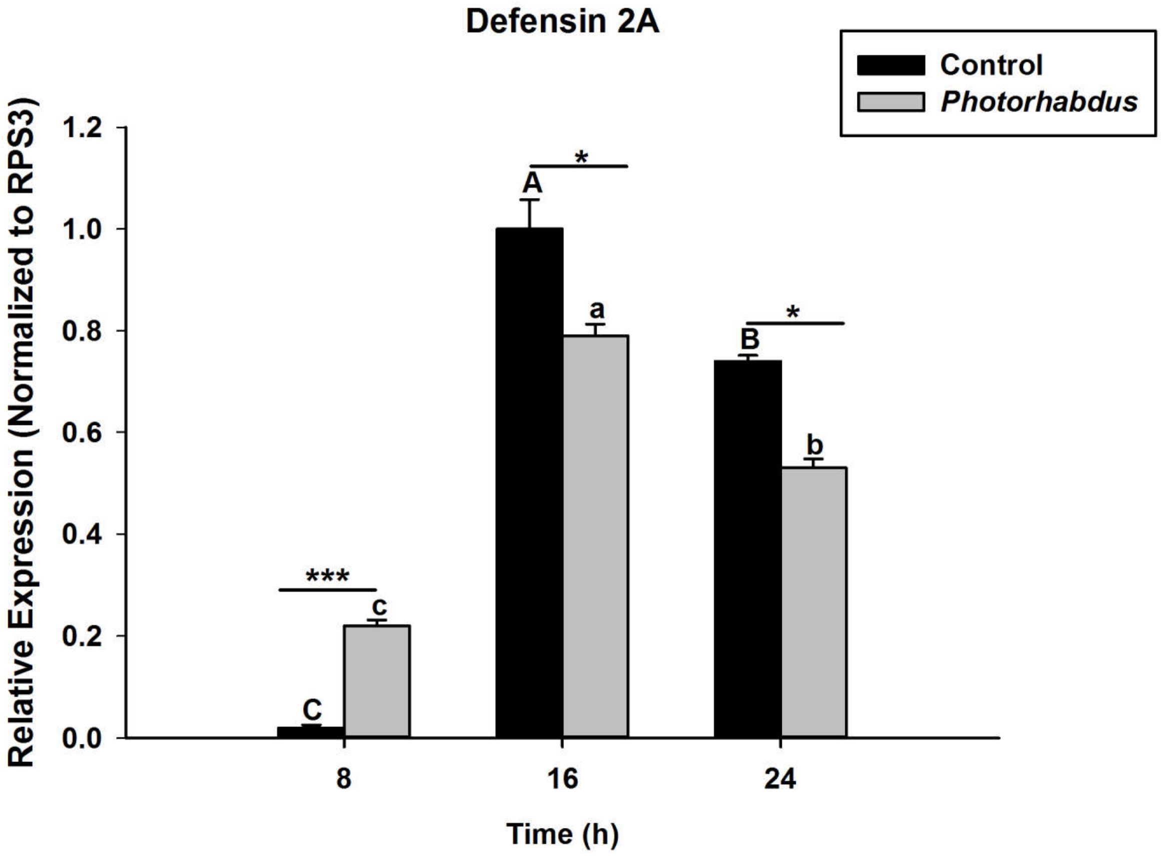

Figure S6: Transcription of antimicrobial peptide (AMP) genes in *O. nipae* larvae infected with *P. luminescens* AMP transcription levels are shown for **Defensin 2A**. Error bars labeled with different letters are significantly different (one-way ANOVA followed by LSD test, $p < 0.05$). The asterisks *** ($P < 0.0001$); ** ($P < 0.001$); * ($P < 0.01$) indicates different significant levels between the control and *P. luminescens* treatments at the indicated time period; while "ns" indicates no significant difference.

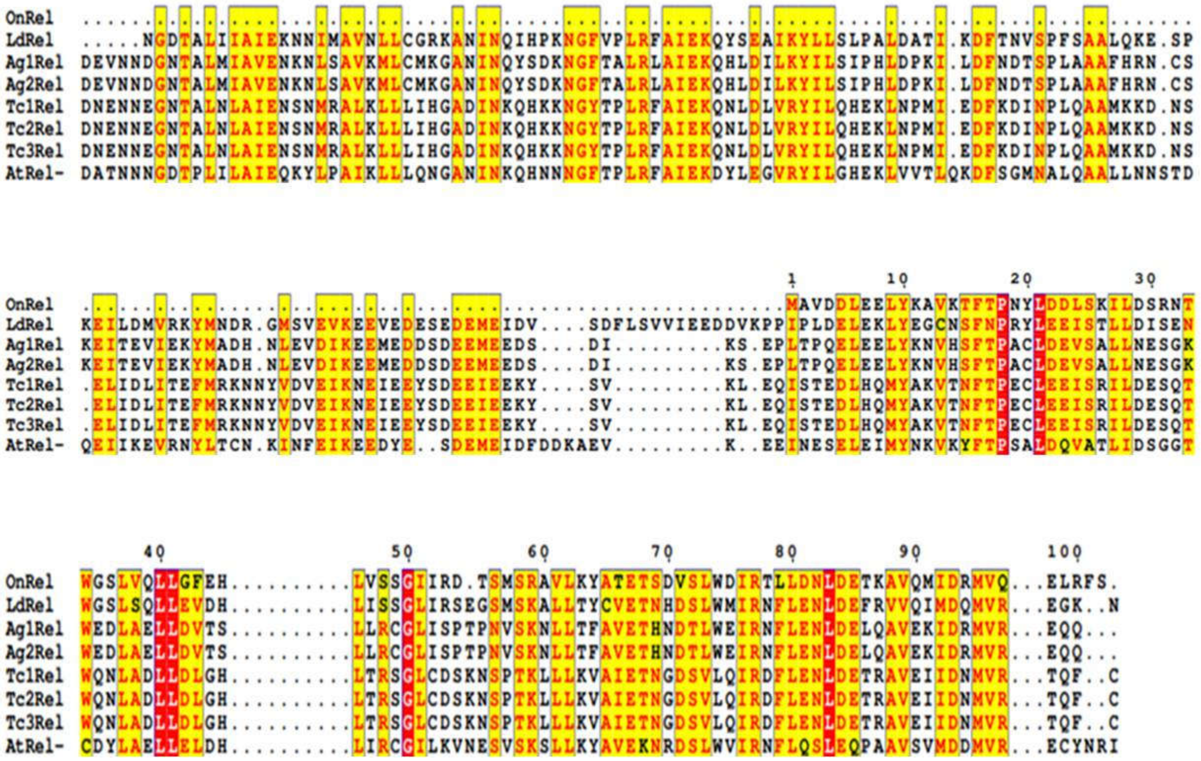

Figure S7: Amino acid sequence alignments of *Relish*/NF-kappa-B p110 subunit-like from O. nipae and other invertebrate: *L. decemlineata* (XM_023174540.1), *A. glabripennis* 1 (XM_018710311.2), *A. glabripennis* 2 (XM_018710307.1), *T. castaneum* 1 (XM_008196263.2), *T. castaneum* 2 (XM_965801.3), *T. castaneum* 3 (XM_965801.3), *A. tumida* 1 (XM_020011061.1) and *L. adelaidensis* (LR134433.1). The alignment was conducted using the ClustalX program and decorated by Espript 3.0 (https://espript.ibcp.fr/ESPript/cgi-bin/ESPript.cgi). Completely conserved residues and conservative substitutions are highlighted in red and yellow, respectively.

111

Table S1: Relish, dsRNA and Antimicrobial Primers used for qPCR

Gene Name	Forward Primer (5’→3’)	Reverse Primer (5’→3’)
q-Relish	GGTTCAACTGCTAGGCTTCG	ACAGGGCTAGGGAAAACTCC
q-Attacin C1	TCCTCGGCATTTGTAGGAGC	TCCGCCATAATGTCTTCCGT
q-Attacin C2	CAAGGGGTTACGGCACTGAT	GGCCGGTCAAATCTACTCCA
q-Attacin C3	GGAGCTGACAGAACGAGAGG	CGGTCACATCAACTCCCACA
q-Defensin 2A	TGTGCTTACCCGTGGATTCA	GCTGCAATGGAAGTTACATGCT
q-Defensin 2B	TGAGCCAGCCAATAGATTTCGA	TTTGAATCCTGCGCCCTCTG
Relish	ATCGATATCAAACCCGAACC	CCATTCTGTCAATCATCTGGA

dsRelish	taatacgactcactatagggACTGCTCTGCCTGGAAGAAA	taatacgactcactatagggAAAAGTCCTTCACCTGGCCT
q-RPS3-F	GACGGTGTCTTCAAAGCTGA	ATTTCTGTACGTGTCGGGGT
